# Establishment of an open database of realistic simulated data for evaluation of partial volume correction techniques in brain PET/MR

**DOI:** 10.1186/2197-7364-2-S1-A44

**Published:** 2015-05-18

**Authors:** Ana Mota, Vesna Cuplov, Jonathan Schott, Brian Hutton, Kris Thielemans, Ivana Drobnjak, John Dickson, Julien Bert, Ninon Burgos, Jorge Cardoso, Marc Modat, Sebastien Ourselin, Kjell Erlandsson

**Affiliations:** 1 Instituto de Biofísica e Engenharia Biomédica, FC-UL, Lisboa, Portugal; 2 Institute of Nuclear Medicine, UCL, London, UK; 3 Centre of Medical Image Computing, UCL, London, UK; 4 INSERM UMR1101, LaTIM, CHRU de Brest, Brest, France

The Partial Volume (PV) effect in Positron Emission Tomography (PET) imaging leads to loss in quantification accuracy, which manifests in PV effects (small objects occupy partially the sensitive volume of the imaging instrument, resulting in blurred images). Simultaneous acquisition of PET and Magnetic Resonance Imaging (MRI) produces concurrent metabolic and anatomical information. The latter has proved to be very helpful for the correction of PV effects. Currently, there are several techniques used for PV correction. They can be applied directly during the reconstruction process or as a post-processing step after image reconstruction. In order to evaluate the efficacy of the different PV correction techniques in brain- PET, we are constructing a database of simulated data. Here we present the framework and steps involved in constructing this database. Static 18F-FDG epilepsy and 18F-Florbetapir amyloid dementia PET/MR were selected because of their very different characteristics. The methodology followed was based on four main steps: Image pre-processing, Ground Truth (GT) generation, MRI and PET data simulation and reconstruction. All steps used Open Source software and can therefore be repeated at any centre. The framework as well as the database will be freely accessible. Tools used included GIF, FSL, POSSUM, GATE and STIR. The final data obtained after simulation, involving raw or reconstructed PET data together with corresponding MRI datasets, were close to the original patient data. Besides, there is the advantage that data can be compared with the GT. We indicate several parameters that can be improved and optimized.

