# Erratum to: Establishment of an open database of realistic simulated data for evaluation of partial volume correction techniques in brain PET/MR

**DOI:** 10.1186/s40658-015-0129-9

**Published:** 2015-11-09

**Authors:** Ana Mota, Vesna Cuplov, Ivana Drobnjak, John Dickson, Julien Bert, Ninon Burgos, Jorge Cardoso, Marc Modat, Sebastien Ourselin, Jonathan Schott, Kjell Erlandsson, Brian Hutton, Kris Thielemans

**Affiliations:** Instituto de Biofísica e Engenharia Biomédica, FC-UL, Lisboa, Portugal; Institute of Nuclear Medicine, UCL, London, UK; Centre of Medical Image Computing, UCL, London, UK; INSERM UMR1101, LaTIM, CHRU de Brest, Brest, France

## Erratum

Unfortunately, the original version of this article [1] contained an error. The author list and author affiliations were included incorrectly. Both of these can be found correctly listed above.
